# High-Fat Diet-Induced Decreased Circulating Bile Acids Contribute to Obesity Associated with Gut Microbiota in Mice

**DOI:** 10.3390/foods13050699

**Published:** 2024-02-25

**Authors:** Haiying Cai, Junhui Zhang, Chang Liu, Thanh Ninh Le, Yuyun Lu, Fengqin Feng, Minjie Zhao

**Affiliations:** 1 School of Biological and Chemical Engineering, Zhejiang University of Science & Technology, Hangzhou 310023, China; caihaiy@hotmail.com (H.C.); liuchang20240220@163.com (C.L.); 2 College of Biosystems Engineering and Food Science, Zhejiang University, Hangzhou 310058, China; zhangjunhui9916@163.com (J.Z.); feng_fengqin@hotmail.com (F.F.); 3 Department of Food Science and Technology, National University of Singapore, Singapore 117542, Singapore; ltninh90@gmail.com (T.N.L.); fstluy@nus.edu.sg (Y.L.)

**Keywords:** obesity, bile acids, lipid homeostasis, gut microbiota, high-fat diet

## Abstract

The altered circulating bile acids (BAs) modulate gut microbiota, energy metabolism and various physiological functions. BA profiles in liver, serum, ileum and feces of HFD-fed mice were analyzed with normal chow diet (NCD)-fed mice after 16-week feeding. Furthermore, gut microbiota was analyzed and its correlation analysis with BA was performed. The result showed that long-term HFD feeding significantly decreased hepatic and serum BA levels, mainly attributed to the inhibition of hepatic BA synthesis and the reduced reabsorption efficiency of BAs in enterohepatic circulation. It also significantly impaired glucose and lipid homeostasis and gut microbiota in mice. We found significantly higher bile salt hydrolase activity in ileal microbes and a higher ratio of free BAs to conjugated BA content in ileal contents in HFD groups compared with NCD group mice, which might account for the activated intestinal farnesoid X receptor signaling on liver BA synthesis inhibition and reduced ileal reabsorption. The decreased circulating BAs were associated with the dysregulation of the lipid metabolism according to the decreased TGR5 signaling in the ileum and BAT. In addition, it is astonishing to find extremely high percentages of taurocholate and 12-OH BAs in liver and serum BA profiles of both groups, which was mainly attributed to the high substrate selectivity for 12-OH BAs of the intestinal BAs transporter during the ileal reabsorption of enterohepatic circulation. This study revealed a significant effect of long-term HFD feeding on the decreased circulating BA pool in mice, which impaired lipid homeostasis and gut microbiota, and collectively resulted in metabolic disorders and obesity.

## 1. Introduction

Obesity has become a very prevalent global health issue among all age populations, which has contributed to the development of metabolic disorders and diseases including non-alcoholic fatty liver, type-2 diabetes, cardiovascular diseases, and cancer [1]. The prevalence of a high-fat diet (HFD) is one of the major contributors to the increasingly obese population, in addition to genetic background, environmental factors and a sedentary lifestyle [2]. Excessive fatness alters normal energy metabolism, physiology and the endocrine milieu. A long-term consumption of a high-fat diet has been linked to insulin resistance [3], leading to increased blood sugar levels, fat storage and hindered fat breakdown. Recent researches suggested that an HFD could significantly alter the composition of gut microbiota, reducing the diversity of gut microbes and the abundance of beneficial microbial species while increasing certain species of Firmicutes and Proteobacteria and harmful metabolites, which collectively result in an impaired gut barrier and disorder of the intestinal function [4]. All these above changes may be associated with systematic inflammation and metabolic disorder, contributing to obesity development.

The critical role of BAs as metabolic regulators has been increasingly emphasized in regulating glucose and lipid metabolism, and energy metabolism [5,6]. Disruption of BA homeostasis has been reported to be associated with various physiological and pathological processes due to their multiple regulation function and potential toxicity [4]. The farnesoid X receptor (FXR) plays an essential role in controlling the feedback inhibition of BA synthesis, BA transport and enterohepatic circulation for homeostasis [7]. According to the previous study, deoxycholate (DCA) is the most effective agonist for FXR, followed by cholate (CA) and chenodeoxycholate (CDCA), whereas lithocholate (LCA) shows a very low FXR-activation effect [8]. Recently, it has been intensively reported that BAs exert important physiological regulation functions through interaction with different cellular receptors, including the nuclear receptors FXR and pregnane X receptor (PXR), and the membrane receptors Takeda G protein-coupled receptor 5 (TGR5) and sphingosine-1-phosphate receptor (S1PR) [9,10]. The BAs receptor TGR5 plays vital roles in regulating energy expenditure and glucagon-like peptide-1 (GLP-1) secretion, and thereby becomes involved in obesity and insulin resistance [11]. It was reported that intestinal-specific FXR deletion could protect mice from obesity and steatosis induced by an HFD [12]. Regulation of intestinal FXR has been used to ameliorate obesity and NAFLD in many studies [13]. The gut microbiota has essential roles in digesting food, training host immunity, regulating gut endocrine function, neurological signaling and metabolic processes [14]. Alteration in BA composition and BA-based signaling is likely influenced by the compositional changes of microbes in the gut, which produce many different BA metabolism enzymes and reshape the BA profile. The genera *Bifidobacterium*, *Lactobacillus*, and *Clostridium* are identified to express high active bile salt hydrolase (BSH) [15,16], which hydrolyzes conjugated BAs to free BAs and probably inactivate intestinal FXR signaling. Administration of caffeic acid phenethyl ester and theabrownin, recognized as inhibitors of bacterial BSH [17,18], displayed the effect of intestinal FXR inhibition and metabolic improvement in HFD-induced obese mice.

The ileal apical sodium-dependent bile acid transporter (ASBT or SLC10A2) is the main transporter that uptakes BAs from the intestinal lumen into intestinal epithelial cells, which is the rate-limiting process for ileal BA absorption [19]. It was reported that ASBT expression was negatively regulated by the FXR-SHP pathway within the ileal cell using an autocrine/paracrine function of FGF15 signaling [5,20]. It was observed that BA malabsorption and alteration in the circulating BA pool occurred in ileal Crohn’s disease patients, which partially accounted for the differences in metabolomics, fecal dysbiosis and inflammatory profiles with other inflammatory bowel disease patients [4]. The influence of carbohydrate components in the diets on serum BA compositions has also been reported recently, and the result showed that the diet with whole grains, legumes, and fruits and vegetables could contribute to modest increases in tauro-lithocholate (TLCA), taurocholate (TCA) and glycocholate (GCA) levels, which might improve glucose homeostasis through acting as ligands for FXR and TGR5 [21]. An HFD can impact BA levels and the metabolism of the host from many aspects. However, the long-term effect of an HFD on BA homeostasis and signaling needs to be further illustrated for the purpose of potential applications of BAs and discovery of new targets for ameliorating obesity caused by an HFD.

With the development of next-generation sequencing and targeted metabolomics technologies, the essential roles of the BAs and gut microbiota in regulating the metabolic process have been gradually revealed [22]. In this study, BA profiles in the liver, serum, ileum and feces of HFD-fed mice with obese phenotypes were analyzed along with normal chow diet (NCD)-fed mice after 16-week feeding. We identified significantly decreased BA levels in liver and serum samples between the HFD and NCD groups, which were partly attributed to the regulation of the BA synthesis pathway and the reabsorption efficiency of BAs in enterohepatic circulation. To our knowledge, this is the first study to reveal the significant decrease in the circulating BA pool caused by long-term HFD feeding in mice. Through the investigation of gut microbiota, we found distinct BSH enzyme activities in ileum microbes between two groups of mice, which were identified to be associated with intestinal FXR signaling. We also investigated the effect of intestinal FXR signaling on BA synthesis and reabsorption in the ileum for BA homeostasis. To determine the regulation effect of the altered circulating BA profile, we also assessed the expression of BA receptor TGR5 and target gene *GLP-1* in the ileum, and UCP1 expression in BAT was conducted. This study revealed a significant effect of long-term HFD feeding on BA synthesis, transporting and the circulating BA pool in mice, which impaired glucose and lipid homeostasis and gut microbiota, contributing to metabolic disorder and obesity in an integrated pattern.

## 2. Materials and Methods

### 2.1. Animal Studies

All mouse studies were approved by the Institutional Animal Care and Use Committee at Zhejiang Chinese Medical University (approval No. IACUC-20201221-02). C57BL/6J mice (five weeks old, 18–20 g weight) were included in this study. To avoid the potential disturbance of sexual hormones, only male mice were used in this study.

These mice were purchased from SLAC Laboratory Animal Co., Ltd., Shanghai, China. The mice were housed in a strictly controlled condition with a 12 h light–dark cycle and a temperature ranging from 22 to 24 °C. The mice had ad libitum access to both water and food. Following a two-week acclimation period, a random assignment was conducted to allocate mice into two groups, with each group consisting of fifteen mice. One group was provided with a chow diet (NCD, Shanghai SLAC Laboratory Animal Co., Ltd., Shanghai, China; 20 kcal% fat, 3850 kcal/kg), and the other group was fed with a high-fat diet (HFD, Jiangsu Medicience Co., Ltd., Yangzhou, China; 45 kcal% fat, 4730 kcal/kg). These mice were administered the diet for a duration of 16 weeks. Table S1 provides a comprehensive composition overview of the two diets, encompassing their specific details. After the completion of the feeding treatment, all the mice were subjected to euthanasia subsequent to a 12 h fasting period. Intestinal contents, feces, serum samples and tissue samples, including liver, ileum, colon, inguinal subcutaneous and epididymal adipose tissues and brown adipose tissues (BAT) were collected and stored at −80 °C until used for analysis.

### 2.2. Histological Analysis

The mice’s epididymal white adipose tissue were preserved in a 10% neutral buffered formalin solution, subsequently embedded in paraffin, and sliced into sections with a thickness of 4 µm. The epididymal adipose tissue was subjected to staining using the hematoxylin and eosin (H&E) technique, following the standard protocols. The liver tissues were stained using oil red O, with imbedding in the optimal cutting temperature to obtain frozen sections at a thickness of 5 µm. These stained slices were analyzed utilizing the Leica Application Suite v4 (Leica Microsystems, Wetzlar, Germany). The dimensions and occurrence of the epididymal white adipocytes and hepatic lipids accumulation were measured using Image-Pro Plus 6.1 software (Media Cybernetics, Rockville, MD, USA).

### 2.3. Biochemical Analysis

The contents of total serum triglycerides (TG), total serum cholesterol (TC), high-density lipoprotein cholesterol (HDLC), and low-density lipoprotein cholesterol (LDLC) were tested using commercial assay kits (Nanjing Jiancheng, Nanjing, China) according to the manufacturer’s instructions. The contents of serum lipopolysaccharide (LPS) and LPS-binding protein (LBP) (Cloud-Clone Corp, Wuhan, China), tumor necrosis factor alpha (TNF-α), interleukin 6 (IL-6), IL-10 and IL-1β (eBioscience, San Diego, CA, USA) were measured by commercial ELISA kits based on the manufacturer’s protocol. Following a period of fifteen weeks of intervention, the mice were subjected to an overnight fasting period. Subsequently, an intraperitoneal glucose tolerance test (IGTT) was conducted, wherein glucose was administered through intraperitoneal injection at a dosage of 2 g/kg body weight. Blood samples were collected from the tail vein, and glucose concentrations were measured using an Accu-Check glucometer (Roche Diagnostics, Basel, Switzerland) at five time points: immediately before and at predetermined intervals (30, 60, 90, and 120 min). The examination of glucose tolerance was used to plot the curve of the IGTT and assess the area under the curve (AUC) using a trapezoidal method. Bile salt hydrolase (BSH) activity of the ileum content was determined by the amount of taurine released from tauro-conjugated bile salt TCA according to Liong and Liu et al. (2019) [23]. A standard curve of BSH activity was prepared based on taurine content in a reaction system. One unit (U) of BSH activity was defined as the amount of enzyme that liberated 1 μmol of taurine from TCA substrate per minute.

### 2.4. Short Chain Fatty Acids Analysis

The measurement of short-chain fatty acids in fecal samples was performed using a Shimadzu GC-2018 system (Shimadzu, Kyoto, Japan), equipped with a flame-ionization detector (FID) and a DB-FFAP column (30 m × 0.53 mm × 0.50 µm; Agilent Technologies Inc., Santa Clara, CA, USA) column in accordance with reference [24]. The fecal samples were weighed and subsequently homogenized in 300 µL of ultra-pure water. The pH of the fecal sample was modified to pH 2, followed by centrifugation at 5000 rcf for a duration of 15 min in order to collect the supernatant. The supernatant was supplemented with 2-ethylbutyric acid as the internal standard, resulting in a concentration of 1 mmol/L.

### 2.5. BAs Analysis

#### 2.5.1. Sample Preparation

Samples of mice liver, serum, ileal contents, and feces weighing 50 mg (or 50 μL) were measured and then mixed with 50 μL of internal standard and 350 μL of extracted solution (methanol: water = 4:1) mixture. Subsequently, the mixture was ground for 6 min (−10 °C, 50 Hz) using a freezer mill, followed by ultrasonication for 30 min (5 °C, 40 kHz), and then incubated at −20 °C for 30 min. After that, the mixture underwent centrifugation at a speed of 13,000 rcf for 15 min at 4 °C in order to collect the supernatant. The entire supernatant was dried with nitrogen and 100 μL of 50% acetonitrile water added for resuspension. After ultrasonication for 10 min (5 °C, 40 KHz), the mixture was centrifuged at 13,000 rcf for 15 min at 4 °C, and the supernatant was collected for analysis. Standard solutions of BAs were generated at a concentration of 1 mg/mL by dissolving each standard in 50% acetonitrile. These solutions were then kept at a temperature of −80 °C. The internal standards (CDCA-D4 and CA-D4) were generated at a proper concentration in methanol and were diluted with the necessary quantity of methanol in order to prepare them for use (2000 ng/mL for CDCA-D4 and 200 ng/mL for CA-D4). A composite of various internal standards (1:1, *v*:*v*) was established for the purpose of sample preparation. A composite of all the chemicals was built in order to introduce the maximum concentration within the calibration curve. A twelve-point calibration curve was prepared and involved the sequential dilution of the working standard. The quality controls were generated by introducing the working standard solution mix into the corresponding samples.

#### 2.5.2. LC-MS/MS Analysis

The quantification of BAs was performed using the AB SCIEX QTRAP 6500+ LC-MS/MS system under the specified chromatographic conditions. The chromatographic separation was performed using a Waters UPLC BEH-C18 column (1.7 μm, 2.1 mm × 150 mm). The mobile phase consisted of two eluents: eluent A, which was composed of water containing 0.1% formic acid, and eluent B, which was acetonitrile containing 0.1% formic acid. The total flow rate of the mobile phase was maintained at 0.4 mL/min. The amount of the injection was 5 uL, and the temperature of the autosampler was adjusted to 50 °C. The electrospray ionization (ESI) and multiple reaction monitoring (MRM) modes were employed for mass spectrometry analysis. The negative ion model was selected and for the ion source setting the pressure of the heating gas (GS1) and the auxiliary heating gas (GS2) were set at 50 pounds per square inch (psi). Additionally, the temperature of desolvation was maintained at 550 °C. The curtain gas pressure (CUR) was recorded at 35 psi, while the collision gas pressure (CAD) was measured at 4 psi. The IonSpray voltage was −4500 V.

### 2.6. Sequencing of the 16S rRNA Microbial Genome and Gut Microbial Diversity

The extraction of bacterial DNA from fecal samples obtained during the 16th week was conducted by the QIAamp DNA Stool Mini Kit (Qiagen, Hilden, Germany). The Illumina MiSeq platform was employed for high-throughput sequencing of the 16S rRNA gene, as outlined in prior studies [25,26]. An amplicon sequencing library was generated by amplifying the V3-V4 region of the 16S rRNA genes using universal primers 341F (5′-CCTACGGGAGGCAGCAG-3′) and 806R (5′-CCTACGGGAGGCAGCAG-3′) via PCR. The PCR amplification product underwent purification and quantification before being subjected to sequencing. The generation of operational taxonomic units (OTUs) involved clustering the sequences with a similarity of over 97% using UPARSE 7.0. This was done to facilitate taxonomical categorization and subsequent analysis. A linear discriminant analysis effect size (Lefse) analysis was performed using an online tool (https://cloud.majorbio.com/ (accessed on 20 September 2023), Majorbio Company, Shanghai, China) with α-index of 0.05 and the threshold of LDA score at 2.0. The resulting data were visualized using an online tool (https://cloud.majorbio.com/ (accessed on 20 September 2023), Majorbio Company, Shanghai, China). To evaluate the influence of two different diet treatments, the averages of microbial compositions were compared to analyze the top 20 abundant genera. The Spearman correlation analysis was conducted to reveal the relationship between the abundance of the critical gut microbes at the genus level and the physiological properties.

### 2.7. RNA Isolation and Quantitative Reverse Transcription PCR

RNA extraction from the homogenized liver was performed using Trizol reagent (Invitrogen, Carlsbad, CA, USA). The concentration and purity of the extracted RNA were afterwards assessed by a Nanodrop ND-2000 spectrophotometer (Thermo Fisher Scientific, Waltham, MA, USA). Subsequently, a quantity of 1μg of RNA was subjected to reverse transcription using a HiScript Reverse Transcription kit (Vazyme, Nanjing, China). The quantitative real-time PCR (qRT-PCR) analysis was performed on a Roche LightCycler 480 instrument (Roche Applied Science, Indianapolis, IN, USA) using a SYBR Color qPCR Master Mix (Vazyme, Nanjing, China). The specific primer sequences used in the experiment are listed in Table S2. The confirmation of the specificity of the amplification products was achieved by the utilization of melting curve analysis. The levels of expression were standardized to GADPH and determined using the 2^−ΔΔCt^ approach.

### 2.8. Statistical Analysis

The data were performed as mean ± standard error of the means (SEM). The statistical analysis was conducted using SPSS 22.0 (SPSS Inc., Chicago, CA, USA) and GraphPad Prism 9.0 (GraphPad Software Inc., San Diego, CA, USA). A one-way analysis of variance (ANOVA) and Tukey’s post hoc multiple comparisons test were applied to examine the statistical disparities across groups. The nonparametric test was conducted using the Kruskal–Wallis test and an unpaired Wilcoxon comparison test. R 2.15.3 was used to perform the Spearman correlation analysis. Statistical significance was determined at *p* < 0.05. The microbiota 16S rRNA sequencing data will be submitted to Genbank of the National Center for Biotechnology Information, which will be publicly available.

## 3. Results and Analysis

### 3.1. HFD-Induced Glucose and Lipid Metabolism Disorder and Obesity in Mice

**Figure 1:**
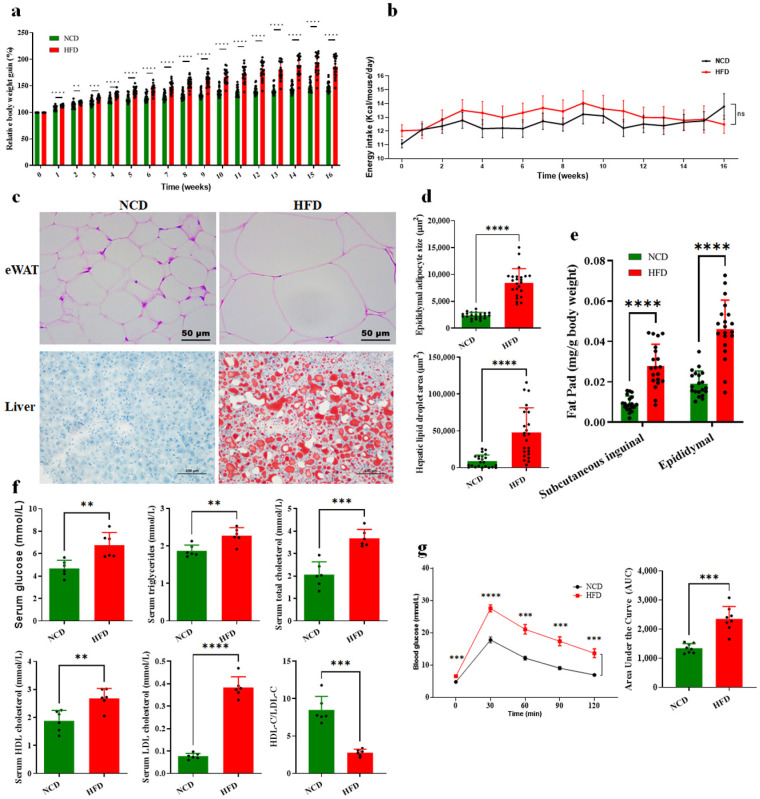
The physiological changes in the HFD and NCD-fed mice for 16 weeks. (**a**) Relative body weights and (**b**) daily energy intake at different time points. (**c**) Representative images of H&E staining of eWAT and oil red O staining of liver sections. eWAT: epididymal white adipose tissue. (**d**) Size of epididymal adipocyte and hepatic lipid droplet areas were assessed using the Image-Pro Plus 6.1 software. (**e**) The ratio of fat pad weight and body weight. (**f**) Serum parameters. (**g**) Intraperitoneal injection glucose tolerance test (IGTT) curve and area under curve (AUC). Data are expressed as mean ± SD. (n = 15 for a and b, n = 24 for d, n = 20 for e, and n = 6 for f and g). **, *p* < 0.01; ***, *p* < 0.001; ****, *p* < 0.0001 (unpaired Student’s *t*-test) compared with NCD group.

The HFD-induced obesity mice model was established to determine the role of the circulating BAs in the regulation of glucose and lipid metabolism. Compared with normal chow diet (NCD)-fed mice, HFD-fed mice showed significantly increased body weight from the 1st week to the 16th week (Figure 1a). However, there were no significant difference in total energy intakes between HFD-fed and NCD-fed groups (Figure 1b). For body fat accumulation, HFD-fed mice showed obvious hypertrophy of epididymal adipocytes and more hepatic lipid droplet accumulation according to the result of histological analysis (Figure 1c,d). Consistently, HFD-fed mice showed significantly increased proportions of subcutaneous inguinal adipose tissue and epididymal white adipose tissue compared to those of the NCD control (*p* < 0.001) (Figure 1e). In addition, the HFD-fed mice had significantly higher serum TG, TC, HDLC, and LDLC levels than the NCD group mice (Figure 1f) and also higher blood glucose content in the examination of glucose tolerance (Figure 1g), suggesting hyperlipidemia and glucose intolerance in HFD-fed mice.

### 3.2. Circulating BA Contents Were Significantly Decreased in HFD-Fed Mice Associated with Obesity

**Figure 2:**
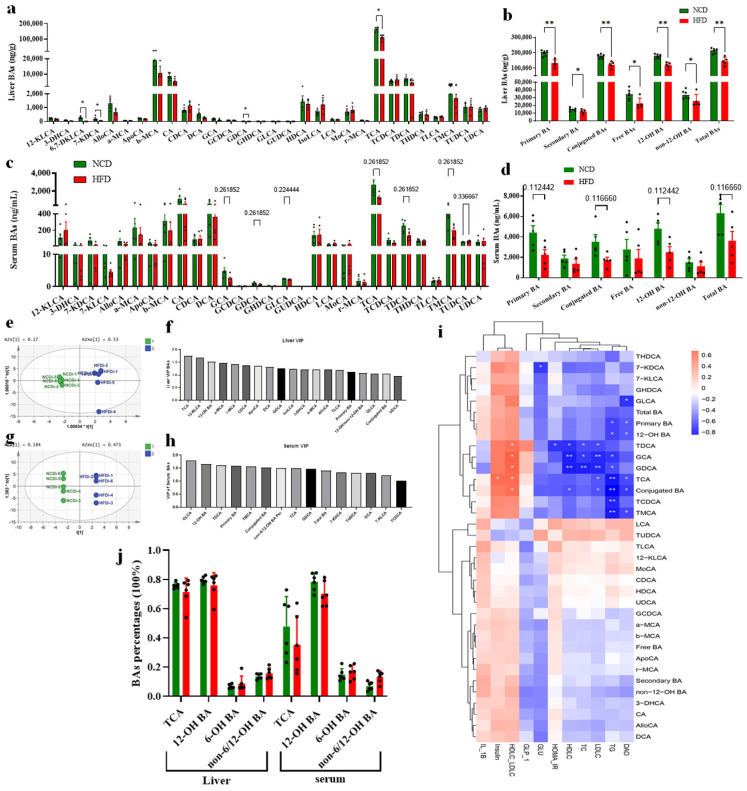
Decreased BA contents in the HFD group. (**a**) Decreased hepatic TCA shown in BA profile in HFD group and (**b**) decreased hepatic total BA. (**c**) Decreased serum TCA shown in BA profile in HFD group and (**d**) and decreased serum total BA. The data are presented as mean ± SD. *, *p* < 0.05; **, *p* < 0.01 (Mann–Whitney U test). (**e**) Orthogonal partial least squares-discriminant analysis (OPLS-DA) scores plot of hepatic BA compositions showing HFD (blue) and NCD (green). (**f**) VIP scores of OPLS-DA based on the hepatic BA profiles between the HFD and NCD groups. A BA with VIP more than 1.0 was considered important in the discrimination between the groups. (**g**) OPLS-DA scores plot of serum BA compositions showing HFD (blue) and NCD (green). (**h**) VIP scores of OPLS-DA using the serum BA compositions between the HFD and NCD groups. (**i**) Heatmap of Spearman correlation coefficients between serum BAs and different parameters (n = 12, 6 samples per group). The gradient colors represent the strength of correlation, with red color indicating strong positive and blue color indicating strong negative. *, *p* < 0.05; **, *p* < 0.01 (Spearman’s correlation with post hoc correction using the Holm’s method). (**j**) High TCA and 12-OH BA percentages in liver tissue and serum composition in HFD and NCD groups. The data are presented as mean ± SD. (Mann–Whitney U test, with no significance).

BAs play a critical role in regulating glycolipid metabolism as signaling molecules. To analyze the BA profile in the HFD and NCD-fed mice, ultra-performance liquid chromatography–quadrupole–time of flight–mass spectrometry (UPLC/Q/TOF/MS) was used to obtain BA profiles of their liver (Figure 2a,b), serum (Figure 2c,d), ileum and feces. Between the NCD-fed group and the HFD-fed group mice, we observed clear separations according to the orthogonal partial least squares-discriminant analysis (OPLS-DA) model established with the BA contents of liver and serum, respectively (Figure 2e,g). The variable importance in projection (VIP) scores from OPLS-DA analysis showed that TCA was the top-ranked BA in liver samples that accounted for the group separation between the HFD and NCD groups (Figure 2f). We found that total BAs, primary BAs, conjugated BAs and 12-OH BAs were significantly (*p* < 0.01) decreased in the HFD group and these differential BAs might be partially attributed to the decreased amount of TCA (Figure 2a). In addition, 7-KDCA, 6,7-DKLCA, secondary BAs, free BAs and non-12-OH BAs also significantly decreased (*p* < 0.05), which indicated that HFD caused a global decrease in BA concentrations in the liver and these decreased circulating BAs in HFD mice might be associated to the obesity phenotype. Similar to BAs in liver samples, the VIP scores from the OPLS-DA model indicated that TCA, 12-OH BAs, and total BAs in serum samples were the traits in BAs which resulted in the group separation between the HFD and NCD groups (Figure 2h). These BAs showed decreased concentrations in the serum samples in the HFD-fed group, though without significance. This result further confirmed the HFD-caused global decrease in circulating BAs in mice. The relation of serum BAs to biochemical parameters was analyzed by Spearman correlation analyses for the HFD and NCD groups. It was observed that most of the differential BAs (TCA, 12-OH BA, conjugated BA) between the HFD and NCD groups were negatively correlated with serum TG, TC and LDLC (Figure 2i). In contrast, most of these differential BAs showed a positive correlation with serum insulin and the ratio of HDLC to LDLC. These results confirmed that HFD caused a global decrease in circulating BAs, which might contribute to the obesity phenotype.

In addition, according to the concentrations of BAs in the liver and serum of the mice fed long term with the HFD and NCD (Figure 2a–d), we were surprised to find that TCA accounted for a very high percentage of the total BA content in the liver of both HFD and NCD-fed mice, 71.44% and 75.78%, respectively (Figure 2j). 12-OH BAs (including TCA, CA, TDCA, DCA, and very few GCA and GDCA) also showed much higher concentrations compared with non-12-OH BAs in the liver of both HFD and NCD-fed mice (Figure 2j). Similarly, serum BAs showed a high percentage of 12-OH BAs in both HFD and NCD-fed mice (70.22% and 78.40%, respectively), which confirmed that the TCA was the most abundant BA species and 12-OH BAs dominated the circulating BA pool in mice fed long term. These results suggested that the BA profiles of the circulating BA pool changed with increasing age, and TCA and 12-OH BAs percentages would increase at least in certain phases, which might be associated with the regulation of BA synthesis and enterohepatic circulation process.

### 3.3. Decreased Circulating BAs Associated with BA Synthesis Regulation

**Figure 3:**
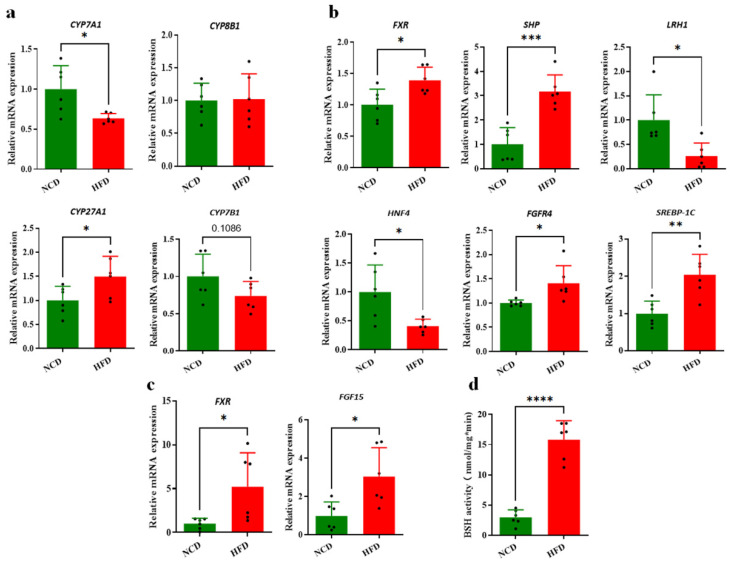
The changes in gene expression related with BA synthesis and regulation caused by HFD. (**a**) Changes in BA synthesis gene expression in HFD group. (**b**) Increased inhibition of BA synthesis based on gene expression related to hepatic FXR and ileal FGF15/FGFR4 signaling. (**c**) Activated ileal FXR/FGF15 signaling based on gene expression. (**d**) Increased ileal BSH activity in HFD group. (n = 6) *, *p* < 0.05; **, *p* < 0.01; ***, *p* < 0.001; **** (unpaired Student’s *t*-test) compared with NCD group.

To determine the role of BA synthesis in altering the BA profile, transcriptional expression of the liver was performed to assess the enzymes expression involved in both classical and alternative BA synthesis. mRNA expression for both 7alpha-hydroxylases CYP7A1 and CYP7B1 was downregulated in the HFD group compared with the NCD group, whereas CYP8B1 expression was not significantly altered (Figure 3a). CYP7A1 is the rate-limiting enzyme of the classical BA synthesis pathway and 12-OH BA synthesis, indicating a decrease in TCA and 12-OH BAs concentrations for the BA pool in the HFD group. Meanwhile, CYP27A1 expression in the HFD group was significantly increased compared with the NCD group (Figure 3a). BA synthesis is mainly regulated by the hepatic FXR/SHP pathway and the intestinal FXR/FGF15 signaling. mRNA expression of *FXR* and *SHP* in the liver was detected to be significantly upregulated in the HFD group compared with the NCD group (*p* < 0.05 and *p* < 0.01, respectively) (Figure 3b), suggesting that the enhanced hepatic FXR/SHP pathway might inhibit BA synthesis in the HFD group. In addition, ileal FXR/FGF15 signaling was investigated and the results showed that FXR and FGF15 were significantly downregulated when expressed in the ileum of the HFD-fed mice (Figure 3c). Accordingly, a significant increase in hepatic FGFR4 expression was identified (Figure 3b), which was induced by the ileal FGF15 through portal vein transport to the liver cell. The mRNA quantification detection showed that HNF4α and LRH1 expression was inhibited in the transcriptional level (Figure 3b), which supported the view that BA synthesis was negatively regulated through hepatic FXR-dependent pathways with the interaction of HNF4α and LRH1.

Activation of FXR in ileum tissue is directly regulated by BA composition and the concentration of ileal contents, so the BA concentrations of ileal contents were analyzed. We found a decrease in conjugated BAs and T-MCA (Figure S1) and a lower ratio of conjugated to free BAs (Figure S2) in HFD group compared with NCD group. It is putative that conjugated BAs including T-βMCA and G-βMCA are antagonists of FXR thus inhibiting the ileal FXR/FGF15 signaling pathway. In addition, BSH activity of the ileum contents was detected to evaluate the hydrolysis ability of ileal microbes in transforming conjugated BAs to corresponding free BAs. HFD-fed mice showed significantly higher BSH activity in their ileal content compared with the NCD group (Figure 3d), which suggested that the HFD caused the gut microbiota alteration in mice and enhanced the BSH-producing species and strains. In conclusion, the HFD enhanced the intestinal FXR/FGF15 signaling through promoting BSH-producing strains and BSH activity, resulting in the inhibition of BA synthesis and decrease in the circulating BA level along with the hepatic FXR/SHP pathway.

### 3.4. Decreased Circulating BAs Resulted from Increased BA Excretion

**Figure 4:**
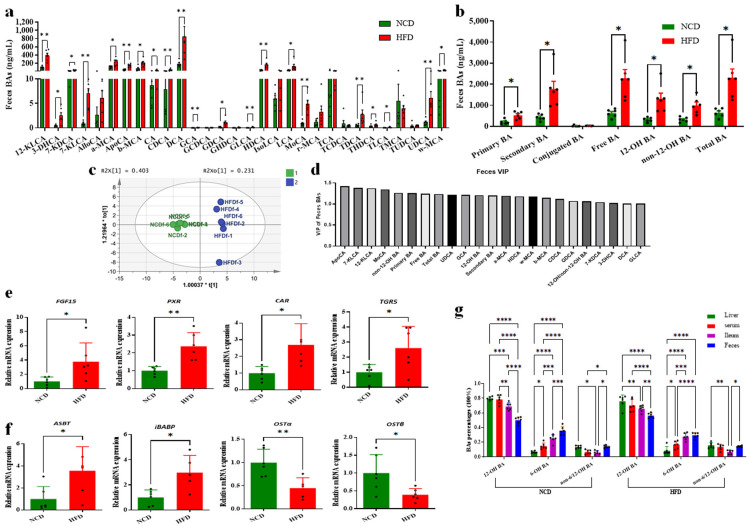
Increased fecal BA contents in the HFD group and selectively distributed 12-OH and non-12-OH BAs. (**a**) Increased fecal BAs shown in BA profile in HFD group and (**b**) increased fecal total BA. The data are presented as mean ± SD (n = 6). *, *p* < 0.05; **, *p* < 0.01 (Mann–Whitney U test). (**c**) Orthogonal partial least squares-discriminant analysis (OPLS-DA) scores plot of fecal BA compositions showing HFD (blue) and NCD (green). (**d**) VIP scores of OPLS-DA according to the fecal BA profiles between the HFD and NCD groups. (**e**) Upregulation of ileal BA receptor gene expression and (**f**) regulation of BA transporter gene expression. Data are expressed as mean ± SD (n = 6). *, *p* < 0.05; **, *p* < 0.01 (unpaired Student’s *t*-test) compared with NCD group. (**g**) Selectively distributed 12-OH, 6-OH and non-6,12-OH BAs in liver, serum, ileum and feces for both HFD and NCD groups. The data are presented as mean ± SD (n = 6). *, *p* < 0.05; **, *p* < 0.01; ***, *p* < 0.001; ****, *p* < 0.0001 (Mann–Whitney U test).

For long-term-fed mice, the alteration in circulating BA pools might be attributed to both BA synthesis regulation and the BA absorption efficiency of the enterohepatic circulation process. To evaluate the efficiency of BA reabsorption of the ileum in mice, we further tested the BA compositions in the feces samples from the HFD and NCD groups. In contrast with BAs in liver, BAs in feces samples of HFD-fed mice showed a global increase compared with the NCD group (Figure 4a,b)**.** Between the NCD- and HFD-fed mice groups, clear separations could be observed according to the OPLS-DA model established with the fecal BAs (Figure 4c). The VIP scores from the OPLS-DA model showed that the top BAs in feces samples included ApoCA, 12-KLCA, non-12-OH BAs, primary BAs, free BAs, total BAs, 12-OH BAs, DCA and so on, which accounted for the group separation between the HFD and NCD groups (Figure 4d). Consistently, most of the detected BAs showed significantly higher concentrations in the HFD group than the NCD group (*p* < 0.05), including DCA (the highest proportion of BA) and 12-KLCA (*p* < 0.01) (Figure 4a). In addition, the primary BAs, secondary BAs, free BAs, 12-OH BAs and non-12-BAs and total BAs were significantly increased. The conjugated BAs showed no significant difference mainly due to the excessive BSH content in the colon of both groups, which transversed nearly all of them to free BAs.

The higher excretion of BAs in the HFD group compared with the NCD group was supported by the significantly higher expression of BA receptors in the colon tissue, including FGF15, PXR, constitutive androstane receptor (CAR), and TGR5 (Figure 4e). To determine the basis of the low ileal fractional absorption rate of BAs in HFD mice, we detected the transcriptional expression of main transporting receptors and proteins. The result showed that mRNA expressions of ASBT and intestinal bile-acid-binding protein (iBABP) were increased (*p* < 0.05), while both organic solute transporting subunits α and β (OSTα and OSTβ) were significantly decreased in the HFD group compared with the NCD group (*p* < 0.01 and *p* < 0.05, respectively) (Figure 4f). The increased expression of BA receptor FXR and BA transporter ASBT suggested that intestinal BA secretion was increased in HFD-fed mice, while the decreased expression of OSTα and OSTβ indicated the ileal BA reabsorption was possibly limited during transporting BAs into the portal vein.

According to the comparison analysis of the percentages of these classes in the samples from the liver, serum, ileum and feces, it was suggested that the extremely high percentages of TCA and 12-OH BAs among the BA profile in both HFD and NCD groups might be attributed to the BA selection reabsorption during the enterohepatic circulation process. To evaluate the selection and efficiency of BA reabsorption of the ileum in mice, we classified all the BAs into 12-OH BAs, 6-OH BAs (MCA, HCA and derivatives) and non-6/12-OH BAs (neither 6-OH nor 12-OH BAs including CDCA, UDCA, LCA and derivatives). The result showed that 12-OH BA percentages were similar between liver and serum samples, but significantly higher than those from ileum and feces samples in both HFD- and NCD-fed mice (*p* < 0.05) (Figure 4g). In contrast, the 6-OH BA percentage from feces samples had the largest percentage, significantly higher than samples from the liver, serum and ileum in NCD-fed mice. In HFD-fed mice, 6-OH BA percentages from feces and ileum samples also displayed significantly higher values than those from the liver and serum (*p* < 0.05). A comprehensive comparison among these classes clearly demonstrated that ileal BAs reabsorption in mice had significant preference for 12-OH BAs rather than 6-OH BAs and non-6/12-OH BAs. In conclusion, the decrease in the circulating BA pool in HFD-fed mice was mainly attributed to the increased BA excretion, repressed BA synthesis and selective reabsorption of 12-OH BAs, which would be tightly associated with the intestinal FXR signaling and BSH-producing gut microbes.

### 3.5. Altered BAs Associated with Gut Microbiota Disorder in HFD-Fed Mice

**Figure 5:**
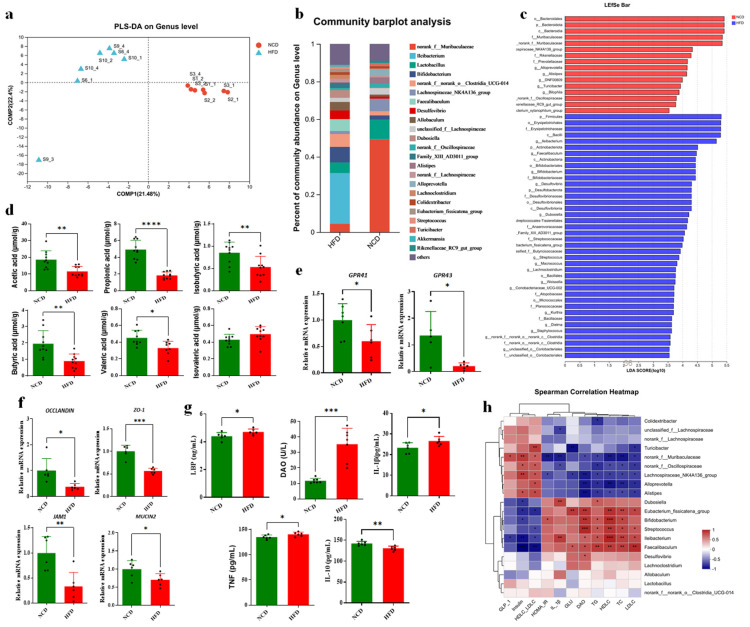
Altered fecal BA compositions associated with gut microbiota. (**a**) Partial least-squares-discriminant analysis (PLS-DA) at genus level identified by 16S rRNA sequencing. PC1 and PC2 account for 21.48% and 22.40%, respectively. n = 7 per group. (**b**) Relative abundance of gut microbiota at genus level in HFD and NCD groups. (**c**) Differentially expressed bacteria based on a linear discriminant analysis effect size (Lefse) method. The NCD group is shown in red and the HFD group is shown in blue. (**d**) The contents of short-chain fatty acids (SCFAs) were significantly decreased in the HFD group (n = 10). (**e**) Downregulated gene expression of SCFA receptors in colon of HFD group. (**f**) Downregulated gene expression of barrier proteins in colon of HFD group. (**g**) Increased inflammation reaction in serum of HFD group. (n = 6). Data are presented as mean ± SD. ** *p* < 0.01 (unpaired Student’s *t*-test). (**h**) Spearman correlations of the inflammation factors with relative abundance of microbial species from the samples of NCD (n = 6) and HFD (n = 6). *, *p* < 0.05; **, *p* < 0.01; ***, *p* < 0.001; ****, *p* < 0.0001 (Spearman’s correlation with post hoc correction using the FDR method).

To determine whether the decreased BA pool and obesity were associated with some key gut microbial strains, 16S rRNA sequencing and analysis of feces samples was performed to compare the microbial compositions of mice between the HFD and NCD groups. The result showed that the α-diversity index including Simpson, Chao1, ACE, and the Shannon index was significantly changed, supporting the theory that gut microbiota could be greatly influenced by diet in mice (Figure S3a). As expected, the relative abundances of Firmicutes, Proteobacteria and Actinobacteria increased in the HFD group while Bacteroidetes decreased dramatically in phyla level compared with the NCD group (Figure S3b). PLS-DA analysis at the genera level was performed, and the result showed that the microbial compositions of feces were obviously separated in the plot between the HFD and NCD groups (Figure 5a). The microbial abundance at genus level is shown in Figure 5b. To identify the alteration in gut microbiota, a linear discriminant analysis effect size (Lefse) method was used in fecal microbial compositions between the HFD and NCD groups (Figure 5c). The results showed that *Ileibacterium* and *Desulfovibrio* were increased in the HFD group, whereas the genus uc_Muribaculaceae and *Lachnospiraceae* NK4A136 groups were decreased (Figure 5b,c). The genera *Ileibacterium* has been reported to be highly present in obesity [27].

Short-chain fatty acids (SCFAs) are crucial metabolites of gut microbes for preserving the energy metabolism balance and regulating host immunity and inflammation as signaling molecules. In this study, most kinds of fecal SCFA content in the HFD group were significantly decreased compared with those in the NCD group (Figure 5d), including acetic acid, propionic acid, butyric acid, isobutyric acid, and valeric acid. Moreover, GPR41 and GPR43, the receptor of SCFAs, were detected to be significantly decreased (*p* < 0.05) for mRNA expression level in the colon (Figure 5e), which confirmed the decreased SCFAs produced by gut microbes. According to the above findings, the gut microbiota disorder caused by HFD and BA alteration resulted in the decreased levels of SCFAs, indicating a potential for gut barrier impairment and a subsequent systematic inflammation risk. The transcriptional expression of tight junction protein Occludin, ZO-1, JAM1 and mucus protein Mucin 2 in the colon were detected, and the result showed all of them were significantly decreased in the HFD group compared with the NCD group (Figure 5f). In addition, lipopolysaccharide binding and protein (LBP) and diamine oxidase (DAO), as the serum markers of the impaired intestinal barrier, were detected to be significantly increased in the HFD group (*p* < 0.05 and *p* < 0.001, respectively) (Figure 5g). We found significant increases in serum TNF-α and IL-1β levels (*p* < 0.05) and a significant decrease in the IL-10 level (*p* < 0.01) in the HFD group, supporting the theory that there is gut microbiota disorder as well as gut barrier dysfunction and a systematic inflammation risk in HFD-fed mice.

The association between the relative abundances of the top 20 fecal microbes and serum biochemical parameters with Spearman correlation heatmap analysis was performed (Figure 5h). It was observed that *Ileibacterium*, *Bifdobacterium*, *Streptococcus* and *Eubacterium fissicatena*_groups displayed a positive correlation with serum glucose content, total TG, TC, LDLC, HDLC and HOMA-IR, while there was a negative correlation with serum GLP-1, insulin and the ratio of HDLC to LDLC, indicating their association with HFD and a high BA concentration environment and a potential contribution to obesity phenotypes. These genera also showed a positive correlation with IL-1β and DAO, supporting the tight association between gut microbiota disorder, gut barrier dysfunction and obesity in HFD-fed mice. *Desulfovibrio* only showed a significant positive correlation with DAO, suggesting its potential role in the dysfunction of the gut barrier. In contrast, the abundance of *uc-Muribaculaceae*, *Oscillospiraceae*, the *Lachnospiraceae*-NK4A136 group, *Alloprevotella* and *Alistipes* were positively correlated with parameters improving metabolic disorder and negatively correlated with TG, TC, LDLC, HDLC and HOMA-IR and inflammation-related factors. The above results suggested that HFD-induced gut microbiota dysbiosis and high BA concentrations in the colon were associated with metabolic disorders and inflammation.

### 3.6. Altered BA Composition Contributed to Obesity in HFD-Fed Mice

**Figure 6:**
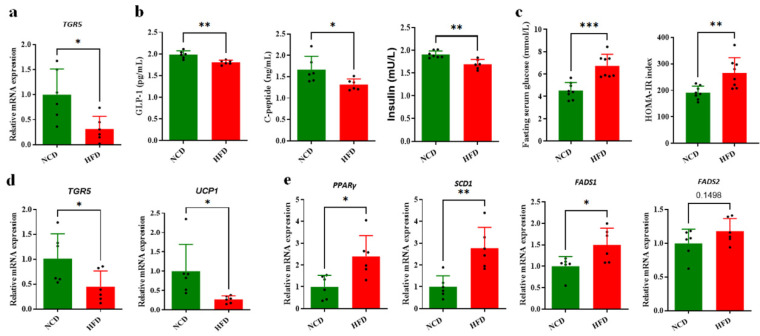
GLP-1 levels in serum and energy expenditure of BAT were significantly decreased in HFD group. (**a**) TGR5 mRNA in ileum was tested using qPCR assay. (**b**) Decreased serum GLP-1 secretion and insulin were identified using ELISA. (**c**) Increased fasting serum glucose concentration was detected using ELISA. (**d**) The mRNA levels of TGR5 andUCP1 in BAT were detected using real-time PCR assay. (**e**) PPARγ-mediated lipid metabolism genes were tested using qPCR assay. Data are expressed as mean ± SD (n = 6). *, *p* < 0.05; **, *p* < 0.01; ***, *p* < 0.001 (unpaired Student’s *t*-test) compared with NCD group.

The expression level of BA receptors and their downstream target proteins that related to glucose and lipid metabolism were detected in different tissues. TGR5 could be activated for glucose and lipid metabolism regulation by many kinds of BAs, with LCA, HCA, DHCA and their conjugated BAs being the most potent natural agonists [11]. In our study, the mRNA expression level of ileal TGR5 was significantly decreased in the HFD group compared with the NCD group (*p <* 0.05) (Figure 6a). In addition, the serum GLP-1, insulin and C-peptide were also decreased significantly in the HFD group (*p < 0*.01, *p <* 0.01 and *p* < 0.05, respectively) (Figure 6b). The fasting glucose concentration was detected to be significantly increased in HFD-fed mice (*p* < 0.05) (Figure 6c), with a significantly increased HOMA-IR value (*p* < 0.01). These results indicated that the decreased circulating BA level in ileum caused by an HFD diet decreased the ileal TGR5 signal, resulting in an alteration in glucose metabolism and increasing risk of obesity and insulin resistance. TGR5 is also highly expressed in BAT, and the increased expression of TGR5 activated by BAs triggers energy expenditure and improves HFD-induced obesity [2]. According to the qPCR detection, the transcriptional expression of TGR5 was significantly decreased in BAT (*p* < 0.05), and UCP1 was also significantly downregulated as a BAT marker in HFD mice (*p* < 0.05) (Figure 6d). These results supported the theory that the downregulated TGR5 signaling regulated by the circulating BA pool in HFD-fed mice decreased the energy expenditure in adipose tissue, partially contributing to obesity and metabolic disorder. A recent study showed that FXRE was present in the promoter of the stearoyl-CoA desaturase (SCD) gene [28], indicating that FXR could directly regulate lipid metabolism through binding to the FXRE of the lipogenesis genes. It was observed that the mRNA expression of *PPARγ* and *SCD* genes was significantly upregulated in the HFD group (Figure 6e), supporting the theory that the abnormally enhanced FXR signaling induced by BAs might be associated with hepatic lipogenesis and lipid metabolism disorders. In addition, the expression level of fatty acid desaturases (FADS1 and FADS2), target genes of PPARγ for lipogenesis, was significantly upregulated in the HFD group (Figure 6e). In summary, the effect of altered circulating BAs caused by the HFD in mice on glucose and lipid metabolism was profound and enduring, which was mainly mediated by the BA receptors FXR and TGR regulation in concert with gut microbiota.

## 4. Discussion

A dramatic decrease in the circulating BA pool was observed in HFD-fed mice according to the quantification analysis on the BA profile of liver, serum, ileum and feces samples in this study, providing new insight into the important roles of BAs in diet-induced obesity and metabolic disorder. Circulating BAs were believed to influence glucose homeostasis and inflammation through activation of FXR and TGR5. We found that the decreased circulating BA level in the ileum caused by the HFD diet impaired ileal TGR5 signaling in mice, which partially accounted for the glucose metabolism disorder and obese phenotypes. In addition, the decrease in BAT thermogenesis of HFD-fed mice was associated with the altered expression of TGR5 and its target genes UCP1 for energy expenditure, contributing to obesity development [29]. Furthermore, overexpression of TGR5 in skeletal muscle cells was also reported to increase energy expenditure [30], suggesting that muscle TGR5 could be influenced by circulating BAs to modulate glucose metabolism and thermogenesis. The TGR5 signaling was identified as being downregulated according to mRNA expression of itself and downstream target genes in the ileum and BAT in the HFD group, which was mainly attributed to the decreased circulating BAs. In addition, it was reported that the tauro-form BAs displayed higher TGR5 activation activities compared with the glycol form and acid form of their corresponding BAs according the tests using TGR5-transfected CHO cells [31]. Therefore, the decrease in conjugated BA in HFD-fed mice in this study also contributed to the alteration in TGR5-mediated regulation on glucose and lipid metabolism.

The nuclear receptor FXR could regulate BA homeostasis, lipid and glucose metabolism. Jiang et al. reported that the intestinal FXR activation caused by an HFD diet in mice was involved in abnormal ceramide synthesis, which directly compromised the beige fat thermogenic function and adverse metabolic phenotypes [32]. Oral administration of FXR antagonists tauro-β-muricholate (T-βMCA) and glycine-β-muricholate (Gly-MCA) prevented or reversed HFD-induced and genetic obesity and metabolic disorder based on reduced biosynthesis of intestinal-derived ceramides [33]. In our study, intestinal BSH activities were detected to be increased in the HFD group compared with the NCD, which was associated with activated intestinal FXR-FGF15 signaling and BA synthesis repression and obese phenotypes. The hepatic FXR also played a role in lipogenesis and fatty acid oxidation. We found that the activated FXR by BAs in the livers of HFD-fed mice increased the *SCD* and *PPARγ* expression associated with hepatic fat accumulation (Figure 6e). In addition, other critical genes in lipid metabolism pathways were significantly up- or downregulated (Figure S4), including *PPARα*, *ACOT1* and *ACOT2* for fatty acid oxidation, *HMGCR*, and *SREBP-1c*. It was indicated that many regulation pathways were involved to promote lipogenesis and obesity besides the BA-related signaling pathway.

The ileal secretion and reabsorption of BAs are critical for maintaining their enterohepatic circulation. We found a dramatic increase in fecal BA concentrations in HFD-fed mice compared with those in the NCD group, indicating a malabsorption of circulating BAs into the ileum cells during enterohepatic circulation. ASBT expression was negatively regulated by intestinal FXR signaling [5]. A previous study indicated that probiotic *L. casei* YRL577 upregulated the mRNA levels of FXR and the fibroblast growth factor 15 (FGF15), whereas it downregulated the mRNA level of ASBT and accelerated cholesterol excretion via defecation [34]. However, intestine-specific *FXR* knockout mice showed no change in ASBT expression while the mRNA levels of other FXR target genes in the ileum significantly decreased compared with wild-type mice fed an HFD [35], indicating that the relationship between ASBT expression and FXR signaling might be environment-dependent. In our study, enhanced signaling of ileal FXR-FGF15 and the high expression of ASBT and iBABP confirmed the increased BA influx in the ileal cell, which indicated that the decreased expression of OSTα/β expression might play a critical role in impeding BA absorption. FXR is the essential regulator for OSTα/β expression, through a fine-tuned dual regulatory pathway mediated by SHP and LRH1 [36]. There might be elaborate cooperation of ASBT and OSTα/β in the BA malabsorption in the ileum regulated by the alteration in BAs and FXR signaling caused by an HFD, which needs to be further researched.

Inadequate synthesis also leads to a smaller pool size in a new steady-state equilibrium with intestinal loss. It is generally agreed upon that intestinal FXR inhibits the bile acid synthesis gene *CYP7A1* based on the FXR-FGF15 signaling regulation, while hepatic FXR is able to modulate primarily the composition of the BA pool by inhibiting *CYP8B1* and *CYP7B1* expression. FGF15 injection in mice significantly inhibited *CYP7A1* expression without influencing *CYP8B1* [37]. It was reported that FGF15 level was significantly reduced in germ free (GF) mice compared with conventionally raised mice, resulting in increased expression of CYP7A1 and higher non-12-OH BA contents. We observed that *CYP7A1* was downregulated in the HFD group compared with the NCD group, which was consistent with the slight decrease in the TCA and 12-OH BAs in the liver and serum. However, expression of the *CYP8B1* and *CYP7B1* genes, encoding two enzymes involved in the classical and alternative BA synthetic pathways, respectively, were reported to be more sensitive to hepatic FXR/SHP-dependent regulation than intestinal FXR signaling [38]. We observed that hepatic *FXR* and its target gene *SHP* were upregulated to express in the HFD group, indicating that hepatic FXR activation was enhanced in mice. Therefore, the downregulated expression of *CYP7B1* was mainly attributed to feedback inhibition of hepatic FXR activation. Taken together, these studies suggest that activation of intestinal FXR/FGF15 and hepatic FXR/SHP signaling caused by HFD in mice generally inhibited BA synthesis, especially via the classic pathway gene *CYP7A1*, which partially accounted for the decrease in the circulating BA pool.

Another astonishing discovery was that both liver and serum BA profiles showed extremely high percentages of TCA and 12-OH BAs in both HFD and NCD-fed mice after 16 weeks of feeding, indicating that the TCA and 12-OH BAs dominated the circulating BA pool in mice fed long term. Based on the novel finding on BA reabsorption selection, we assumed that the high accumulation of TCA and 12-OH BAs in the liver in both the HFD and NCD groups was mainly attributed to a higher fractional absorption rate (FAR) of 12-OH BAs in the repeated enterohepatic circulation for mice fed long term, whereas in mice fed short term in a previous study, a close proportional ratio of 12-OH BAs and non-12 BAs was observed [2]. In contrast, we have observed a similar high percentage of TCA and 12-OH BAs in mice with 26 weeks’ feeding according to the figures provided from a previous study [18]. These results suggested that BA profiles of the circulating BA pool might change with increasing age, and TCA and 12-OH BAs percentages would increase at least in certain phases based on the reabsorption selection of special BAs during the repeated enterohepatic circulation process. 6-OH BAs includes HCA, HDCA, and T-βMCA, which are antagonists of FXR [11], as the ratio of 12-OH BAs to 6-OH BAs increases with aging, implying signaling enhancement of FXR regulation and a potential risk of subsequent metabolic disease and obesity. It was found that insulin resistance was correlated with increased serum 12-OH BAs in both nondiabetic and diabetic individuals [39]. A study reported that targeted knockout of the *CYP8B1* gene in mice resulted in a complete absence of CA and 12-OH BAs and improved glucose tolerance, insulin sensitivity, and β-cell function [40]. Our study found a different pattern for increased serum 12-OH BAs associated with age in mice mainly based on the intestinal reabsorption selection during the repeated enterohepatic circulation process, which might provide new insight into the altered effect of BA signaling on insulin resistance and metabolic disorder with age. It was concluded that ASBT showed similar affinities between free BAs and their corresponding conjugated forms [41]. Previous studies also found that CDCA and its conjugated forms showed a higher affinity to ASBT than DCA and CA species, supporting the theory that the BA selectivity of ASBT might be related to special BA species accumulation in the liver and serum in our study.

Through the investigation of gut microbiota by 16S rRNA sequencing, we found significantly increased BSH activity in ileum microbes between two groups of mice, which were identified as being associated with intestinal FXR signaling, BA homeostasis and other host metabolisms [17,18]. A recent study also reported that the BA substrate specificity of BSH also plays an important role in regulating BA synthesis and altering the BA composition [42]. Therefore, the precise relationship of BSH activity and the specificity of BAs with key microbial species should be illustrated for further precision regulation of gut microbe and FXR-based bile acid synthesis, leading to beneficial physiological effects. The correlation of the relative abundances of fecal microbes and fecal BA content with Spearman correlation was conducted (Figure S3d), which might be helpful for understanding BA metabolism and tolerance of gut microbes. The heatmap of hierarchical cluster analysis (HCA) showed that the gut microbes were generally divided into three clades according to their correlation relationships with BA composition, whereas the BAs were classified into conjugated BAs and free BAs, correspondingly. The first clade of gut microbes, including *Bifdobacterium*, *Ileibacterium* and *Desulfovibrio*, displayed positive correlations with both kinds of BAs and total BAs as well, indicating their strong capacity for tolerating and hydrolyzing conjugated BAs to free BAs with produced BSH enzyme. This clade might be further classified based on the different bio-transformation direction of secondary BAs mediated by the differences in activity and selectivity of microbial BA dehydroxylases and isomerases. On the contrary, the gut microbes in the second clade negatively correlated to most BAs, suggesting their sensitivity to BA components. The third clade showed a relatively positive correlation with conjugated BAs while there was a negative correlation with free BAs and secondary BAs, suggesting that they had low or no BSH activity like the second clade. However, the third clade might tolerate higher concentrations of conjugated BAs compared with the second clade, due to other mechanism for BA tolerance, or they could selectively hydrolyze non-12-OH BA substrates rather than 12-OH BAs. These results suggested that gut microbiota dysbiosis and fecal BA alteration may impose a substantial impact on each other, which influences the gut barrier, systematic inflammation and metabolic health in a kinetic cross-talking manner. On one side are gut microbiota evolved to the gut environment based on their capacity of tolerating and bio-transforming BAs [43]. On the other side, BA synthesis and enterohepatic circulation were regulated based on the ileal FXR-FGF15 signaling pathway. BSH-producing strains were emphasized for producing free BAs by hydrolyzing the conjugated BAs [44], which was thought to be the critical step to activate the ileal FXR. Due to the tight interaction with gut microbe and host, the importance of BA should be underscored in food digestion, energy metabolism, gut microbiota and barrier, inflammation and other aspects.

To summarize, based on the fundamental regulation function of FXR in mRNA expression of BA synthesis, transporting proteins and receptor genes, the activated FXR in HFD-fed animals may play an essential role in suppressing the feedback inhibition of BA synthesis, BA transport and enterohepatic circulation for homeostasis disruption. This dietary factor will eventually lead to an alteration in circulating BAs and metabolic disorder. In this study, it was found that altered FXR-related BA signaling mediated by gut microbiota was associated with a significant decrease in the circulating BA pool, which contributed to a dysregulated host metabolism and obese phenotype. Therefore, it would be a promising strategy to precisely regulate circulating BAs for obesity therapy caused by HFD based on construction and modulation of the critical interaction between gut microbiota, circulating BAs and metabolic regulation.

## Data Availability

The original contributions presented in the study are included in the article/supplementary material, further inquiries can be directed to the corresponding author.

## Supplementary Materials

The following are available online at https://www.mdpi.com/article/10.3390/foods13050699/s1, Figure S1: Decreased conjugated BAs and T-MCA in the ileum contents of HFD and NCD group. (a) Decreased ileal T-MCA shown in BA profile in HFD group and (b) decreased ileal conjugated BAs. The data are presented as the mean ± SD. No significance (Mann-Whitney U test). (c) Orthogonal partial least squared-discriminant analysis (OPLS-DA) scores plot of ileal BA profiles showing the groupings of HFD (blue) and NCD (green). (d) VIP scores of OPLS-DA based on the ileal BA profiles between the HFD and NCD group. A BA with VIP more than 1.0 was considered important in the discrimination between the groups; Figure S2: Ratios of different BA classification in HFD and NCD group. (a, b, c, d) Ratios of primary BA to secondary BA, conjugated BA to free BA and 12-OH BA to non-12-OH BA in liver, serum, ileum content and feces, respectively. Data are expressed as mean ± SD (n = 6). No significance (unpaired Student’s t-test) compared with NCD group.; Figure S3: Gut microbiota were regulated by HFD and associated with fecal BA in mice. (a) The α-diversity index including Simpson, Chao1, ACE, and Shannon index was significantly influenced by HFD in mice. (b) Significantly changed microbial species in HFD group according to Wilcoxon rank-sum test bar plot on phyla level compared with NCD group and (c) on genus level. (d) Spearman correlations of the relative abundances of fecal microbes with fecal BA content from the samples in the group of NCD (n = 6) and HFD (n = 6). The gradient colors represent the strength of correlation, with red color indicating strong positive and blue color indicating strong negative; Figure S4: Other critical genes in lipid metabolism pathways were significantly up- or down-regulated. Table S1: Compositions of experimental diets; Table S2: The primers used in this study.
