# Supplementary material for: High-Fat Diet-Induced Decreased Circulating Bile Acids Contribute to Obesity Associated with Gut Microbiota in Mice
Source: Foods. 2024 Feb 25;13(5):699. doi: 10.3390/foods13050699 (PMC10931208; doi:10.3390/foods13050699)

Figure S1 Decreased conjugated BAs and T-MCA in the ileum contents of HFD and NCD group. (a) Decreased ileal T-MCA shown in BA profile in HFD group and (b) decreased ileal conjugated BAs. The data are presented as the mean ± SD. No significance (Mann-Whitney U test). (c) Orthogonal partial least squared-discriminant analysis (OPLS-DA) scores plot of ileal BA profiles showing the groupings of HFD (blue) and NCD (green). (d) VIP scores of OPLS-DA based on the ileal BA profiles between the HFD and NCD group. A BA with VIP more than 1.0 was considered important in the discrimination between the groups.

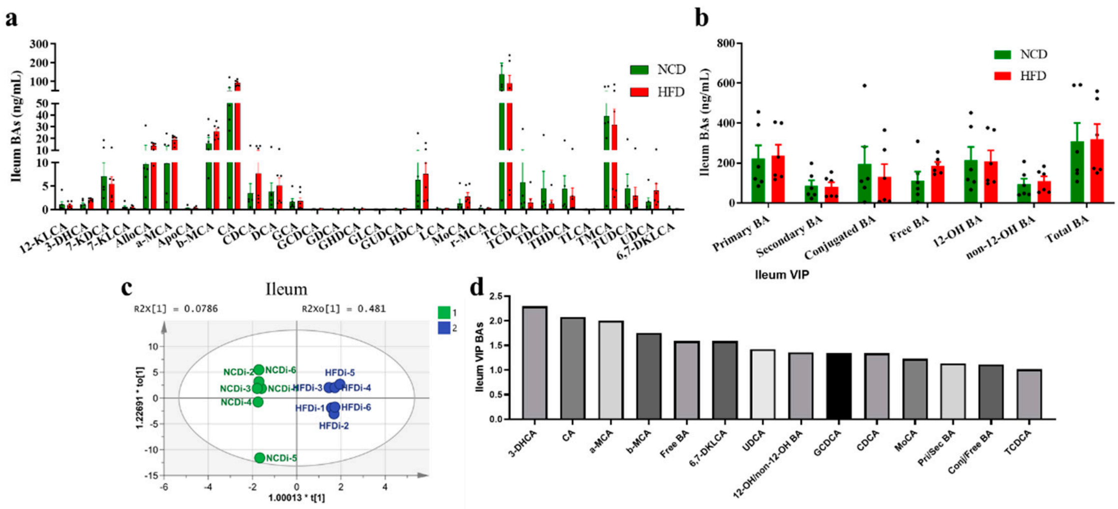

Figure S2 Ratios of different BA classification in HFD and NCD group. (a, b, c, d) Ratios of primary BA to secondary BA, conjugated BA to free BA and 12-OH BA to non-12-OH BA in liver, serum, ileum content and feces, respectively. Data are expressed as mean ± SD (n = 6). No significance (unpaired Student's t-test) compared with NCD group.

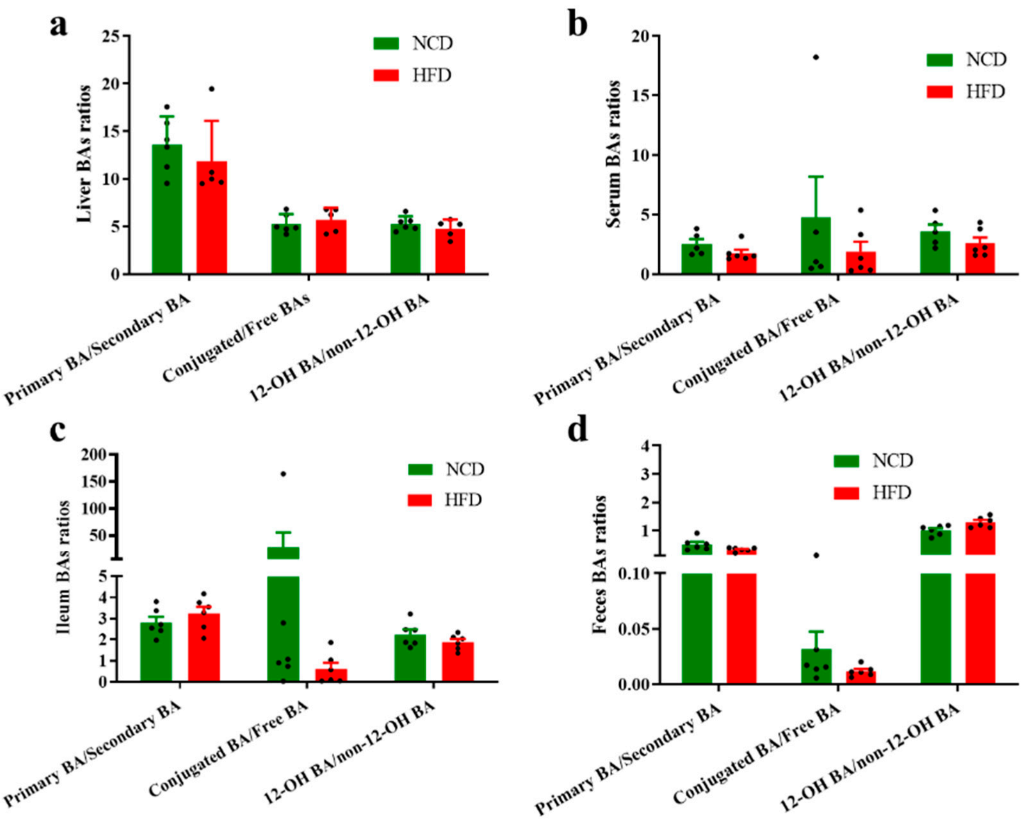

Figure S3 Gut microbiota were regulated by HFD and associated with fecal BA in mice. (a) The α-diversity index including Simpson, Chao1, ACE, and Shannon index was significantly influenced by HFD in mice. (b) Significantly changed microbial species in HFD group according to Wilcoxon rank-sum test bar plot on phyla level compared with NCD group and (c) on genus level. (d) Spearman correlations of the relative abundances of fecal microbes with fecal BA content from the samples in the group of NCD (n = 6) and HFD (n = 6). The gradient colors represent the strength of correlation, with red color indicating strong positive and blue color indicating strong negative. *, $P < 0.05$; **, $P < 0.01$ (Spearman's correlation after the post hoc correction using the FDR method).

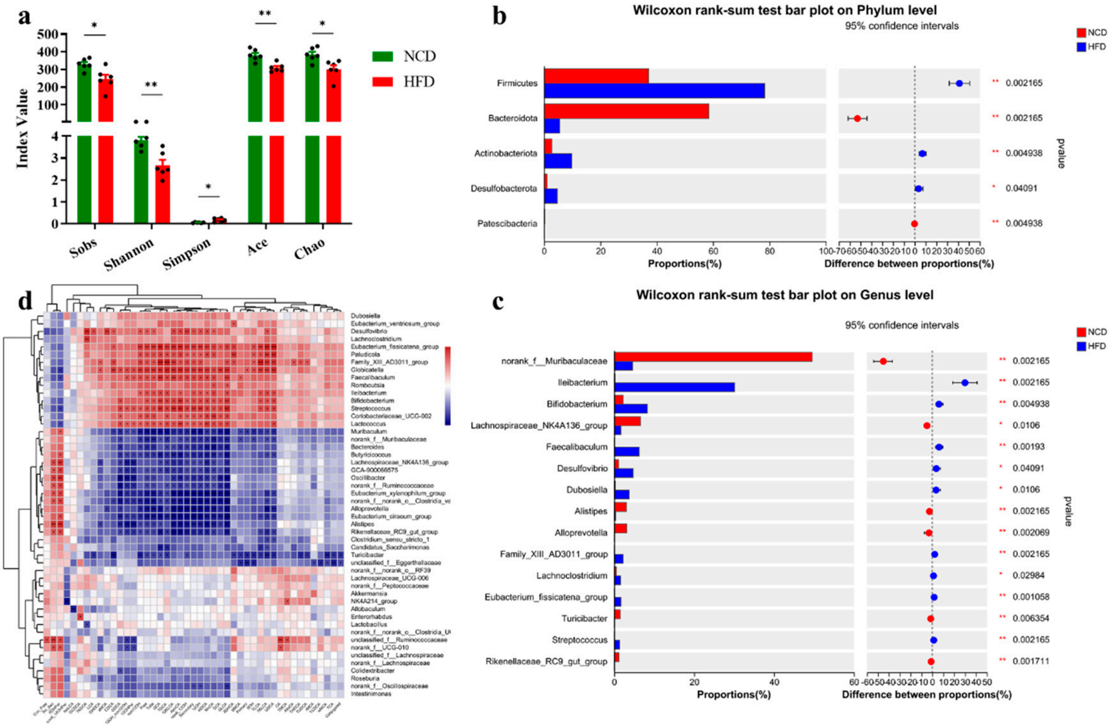

Figure S4 Other critical genes in lipid metabolism pathways were significantly up- or down-regulated. Data are expressed as mean ± SD (n = 6). *, $P < 0.05$; **, $P < 0.01$; ***, $P < 0.001$ (unpaired Student's t-test) compared with NCD group.

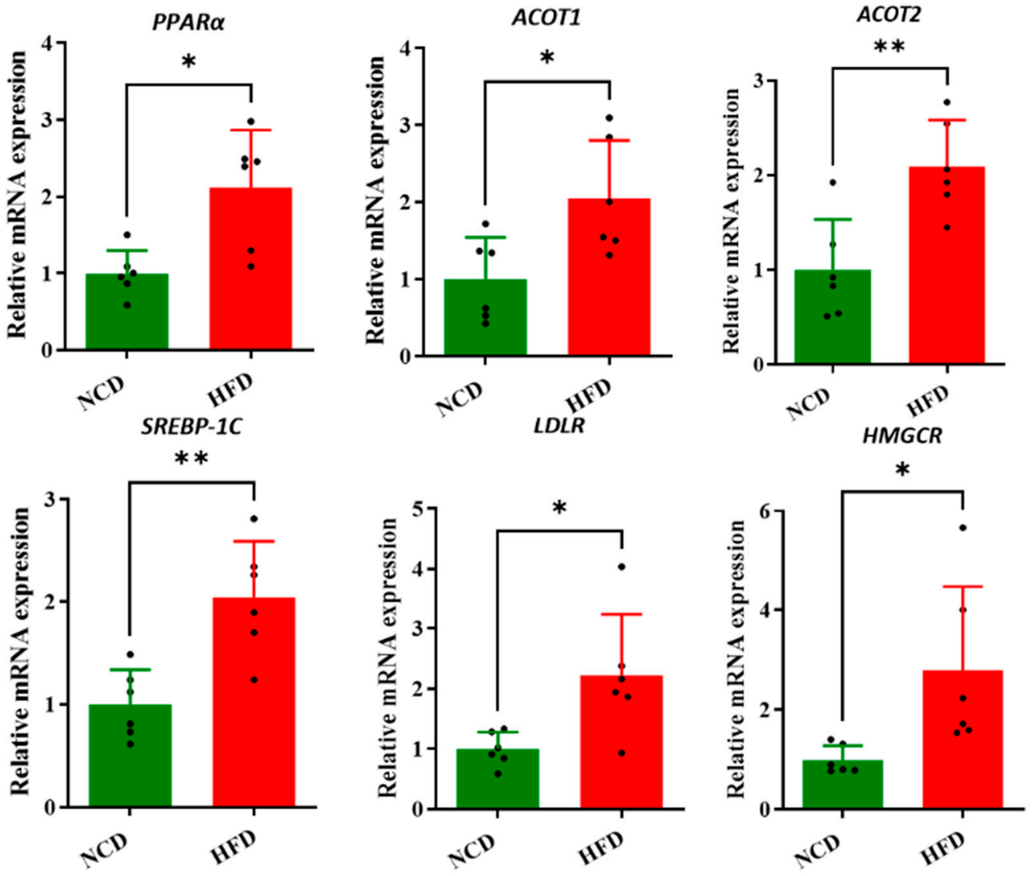

Supplement: Supplementary file 1 [file foods-13-00699-s001.zip › Supplementary material Figures new.pdf]
