# Supplementary material for: High-Fat Diet-Induced Decreased Circulating Bile Acids Contribute to Obesity Associated with Gut Microbiota in Mice
Source: Foods. 2024 Feb 25;13(5):699. doi: 10.3390/foods13050699 (PMC10931208; doi:10.3390/foods13050699)
Supplement: Supplementary file 1 [file foods-13-00699-s001.zip › Supplementary material Table S1.pdf]

Supplementary Table S1. Compositions of experimental diets

Ingredients (g / 100 g diet)	Normal chow diet	High fat diet
Casein	18.96	23.31
L-Cystine	0.28	0.35
Corn Starch	29.86	8.48
Maltodextrin	3.32	11.65
Sucrose	33.17	20.14
Cellulose	4.74	5.83
Soybean Oil	2.37	2.91
Lard	1.90	20.68
Mineral Mix	2.68	3.31
Potassium Citrate, 1 H_2O	1.56	1.92
Vitamin Mix	0.95	1.16
Choline Bitartrate	0.19	0.23
**Calories supplementation (kcal %)**		
Proteins	20	20
Carbohydrates	70	35
Fats	10	45
**Total calories (kcal / 100 g diet)**	385	473
