# Supplementary material for: High-Fat Diet-Induced Decreased Circulating Bile Acids Contribute to Obesity Associated with Gut Microbiota in Mice
Source: Foods. 2024 Feb 25;13(5):699. doi: 10.3390/foods13050699 (PMC10931208; doi:10.3390/foods13050699)
Supplement: Supplementary file 1 [file foods-13-00699-s001.zip › Supplementary material Table S2.pdf]

Supplementary Table S2. The primers used in this study

Name	Sequence (5'-3')
TNFα Forward	AGGCACTCCCCCAAAAGAT
TNFα Reverse	CAGTAGACAGAAGAGCGTGGTG
PPARa Forward	GTCCTCAGTGCTTCCAGAGG
PPARa Reverse	GGTCACCTACGAGTGGCATT
PPARγ Forward	GCATTTCTGCTCCACACTATGA
PPARγ Reverse	TCGCACTTTGGTATTCTTGG
srebp1 Forward	GATCAAAGAGGAGCCAGTGC
srebp1 Reverse	TAGATGGTGGCTGCTGAGTG
SCD1 Forward	CCGAAGAGGCAGGTGTAGAG
SCD1 Reverse	TTCTTACACGACCACCACCA
HMGCR Forward	GGACCAACCTTCTACCTC
HMGCR Reverse	CCATCACAGTGCCACATAC
FXR Forward	CTGACCCTGCCATCCAAGT
FXR Reverse	TCAGAAGCCGATGTTCCTCT
SHP Forward	GGAGTATGCGTACCTGAAG
SHP Reverse	TGCCTGGAATGTTCTTGAG
HNF4 Forward	GGATATGAGGAGGCTATACTG
HNF4 Reverse	GAAGGTTCTATGCGATGGT
LRH1 Forward	GCTACTTCTTCGGCTACC
LRH1 Reverse	CGTCCTCCTCTTCTCCTT
TGR5 Forward	GTTATCGCTCATCTCATTGG
TGR5 Reverse	TTGGCTACTGGTGTGGTA
FGF15 Forward	GCCATCAAGGACGTCAGCA
FGF15 Reverse	CTTCCTCCGAGTAGCGAATCAG
FGFR4 Forward	CTCCTTAACCTCCATCAGTAA
FGFR4 Reverse	TGCCTCCAATACGATTCTC
PXR Forward	GGACATACTGATAGCCAACA
PXR Reverse	CCACCTAACACCTGAGAATT
CAR Forward	GCATATCTCACTCAACACTAC
CAR Reverse	CGCCATCTCCTCTTGTAG
ASBT Forward	CCAATATCCTGGCCTATTGGAT
ASBT Reverse	AGAGCAACCAGAGAAATACCAA
iBABP Forward	TTCAAGATCATCACAGAGGTCC
iBABP Reverse	CATGGTCTGCATTTCACATTCT
OSTα Forward	TTCCGTCAAGCCAAGATG
OSTα Reverse	CAGCGAACAAGCCTCATA
OSTβ Forward	CGGCTCCTTGGAATTATTC
OSTβ Reverse	CCTGGCTGTTGTTCTCTT
Occluding Forward	GTGAGCTGTGATGTGTGTTGAGCT
Occluding Reverse	GTGGGGAACGTGGCCGATATAATG
Mucin2 Forward	AAGTACAGATCAAGACCGTGAGG

Mucin2 Reverse	CCACTAACTGCTTGTTCACCTG
Cyp7a1 Forward	TGGGCATCTCAAGCAAACAC
Cyp7a1 Reverse	TCATTGCTTCAGGGCTCCTG
CYP27A1 Forward	GACACTGCCGCCTTCATC
CYP27A1 Reverse	GCCATTCAGGTATCGCTTCC
CYP8B1 Forward	CAGCCTATCCTTGGTGATG
CYP8B1 Reverse	TCCTTGGTGTAGCCGAAT
CYP7B1 Forward	GCTTACTGATGACGACCTT
CYP7B1 Reverse	TTCTGTGTTCCAATCTGTGA
UCP1 Forward	AGGCTTCCAGTACCATTAGGT
UCP1 Reverse	CTGAGTGAGGCAAAGCTGATTT
GPR41 Forward	AGTGCCAGTTGTCCAATACTCT
GPR41 Reverse	AAGAAGCTTGTCCCCATGGTC
GPR43 Forward	CAATCAGAAGACAGAAAAGGAGCTG
GPR43 Reverse	GTCTGGGGTCATTCTCCTTGG
